# Effects of Cocaine and Fasting on the Intake of Individual Macronutrients in Rats

**DOI:** 10.3389/fnins.2019.00805

**Published:** 2019-08-02

**Authors:** Nia Mitchell, Aaron G. Roseberry

**Affiliations:** ^1^Department of Biology, College of Arts and Sciences, Georgia State University, Atlanta, GA, United States; ^2^Neuroscience Institute, College of Arts and Sciences, Georgia State University, Atlanta, GA, United States

**Keywords:** cocaine, feeding, fasting, carbohydrate, protein, fat

## Abstract

Obesity is a significant problem, and increased food intake is thought to underlie much of the increase in obesity levels. Recently, there has been much discussion and debate about the role of the individual macronutrients, carbohydrates, fat, and protein, in the rise in obesity levels and its associated comorbidities, but overall there has been little study of how different treatments and stimuli that affect feeding impact the intake of individual macronutrients. In these studies, we tested whether two treatments leading to altered feeding, acute cocaine injection and an acute fast, differentially affect the intake of individual macronutrients using a three diet choice paradigm. Cocaine strongly inhibited the intake of each individual test diet (carbohydrate, fat, and protein), but there were no differences between its effects on the intakes of each individual diet. In contrast, an acute fast had little effect on the intake of any of the diets and did not differentially affect the intake of the three test diets. Thus, these studies demonstrate that cocaine can effectively inhibit the intake of feeding independent of its macronutrient content, and significantly advance our understanding of the neural regulation of individual macronutrient intake.

## Introduction

Obesity has become a significant problem in the United States and is growing worldwide. Approximately 70% of the adult population can be considered overweight or obese (Flegal et al., 2010, 2016) with an estimated cost in excess of $2 billion annually (Finkelstein et al., 2009; Cawley and Meyerhoefer, 2012). Although changes in activity likely have contributed to the increases in obesity levels, this rise is thought to be due primarily to increases in food intake. There are currently few effective treatments available to reduce food intake, however. Thus, we need a better understanding of the mechanisms controlling feeding so new approaches can be identified to combat obesity.

The composition of food alters its appeal and its qualities. For example, foods that are high in fat and/or sugar are highly appetizing and palatable, which increases the rewarding qualities of these high fat/high sugar foods. There is currently significant debate about the contribution of individual macronutrients (fat vs. carbohydrate vs. protein) to the rise and development of obesity and its accompanying complications (e.g., Hall, 2018). For example, there has been increasing attention to the potential role of added sugars in the rise in obesity levels (Wiss et al., 2018), and studies have shown that diets high in sugars can lead to obesity, diabetes, and addictive/compulsive-like feeding in animal models (Colantuoni et al., 2001, 2002; Johnson and Kenny, 2010; Wiss et al., 2018). In contrast, research animals strongly prefer foods high in fat and a recent study comparing the effects of diet composition on weight gain and obesity development demonstrated that body weight gain was directly related to the amount of fat consumed with no relation to the sugar, carbohydrate or protein content of the food (Hu et al., 2018). Thus, although the fat and sugar content of food appears to play an important role in its palatability and likely contributes to its intake and to weight gain, it is still unclear how the intakes of individual macronutrients contribute to overall feeding and the development of obesity.

Despite the evidence showing that individual macronutrients can significantly affect feeding and can contribute to the development of obesity, there has been little study of how different treatments that alter feeding affect the intake of individual macronutrients. In these studies we tested whether two treatments that acutely alter feeding, short-term fasting or an acute cocaine injection, differentially alter the intake of individual macronutrients using a short-term (24 h) three diet choice test.

## Materials and Methods

### Reagents

Sterile bacteriostatic saline was from Patterson Veterinary Supply, Inc., (Sterling, MA, United States). Cocaine HCl was from Sigma. All other reagents were from common commercial sources.

### Animals

Young adult male Sprague Dawley rats (Harlan Laboratories, Madison, WI, United States) were used for these experiments. Rats weighed between 220–250 g upon arrival (∼7–8 weeks old) and were ∼16–19 weeks old upon testing. Rats were individually housed in ventilated polycarbonate Animal Care System cages in a temperature- and humidity-controlled room on a reverse 12:12 light/dark cycle with *ad libitum* food and water, except for acclimation and testing days, when the rats had access to the individual macronutrient choice diets. All protocols and procedures were approved by the Institutional Animal Care and Use Committee at Georgia State University and conformed to the National Research Council of the National Academies *Guide for the Care and Use of Laboratory Animals.*

### Test Diets and Measurement of Food Intake

Rats had *ad libitum* access to water and standard laboratory chow (Purina rodent diet #5001, PMI Nutrition International; 3.36 kcal/g) in their home cage, with the exception of all acclimation and test days. To test the intake of each individual macronutrient (carbohydrate, fat, or protein), three different diets (hereafter referred to as the test diets) that contained only a single macronutrient combined with essential vitamins and minerals were prepared. The composition of the diets were adapted from Smith et al. (1996) and are shown in Table 1. Prior to the start of the experiment, rats were acclimated to the test diets for 24 h on multiple trials spaced at least 3 days apart until the intake of each diet was stable. Stable intake was defined as < 10% variation in the average intake of each individual diet over 3 consecutive tests and a lack of increase in the mean intake during at least one of the 3 stable acclimation tests. Diet intakes during the acclimation tests are shown in Table 2. Pre-weighed amounts of each diet were given to the rats in petri dishes magnetically adhered to the bottom of their home cage to limit spillage, and the dishes were weighed at specific intervals to determine the amount eaten. If a food dish was spilled, it was replaced with a new set amount of food at the next time point, and the data for the prior time period was excluded. 24 h chow intake was monitored periodically throughout the experiment and during the 24 h post-test period by providing a set amount of food in the normal food hopper, which was then weighed 24 h later.

**TABLE 1:** Composition of test diets.

**Ingredient**	**Carbohydrate**	**Fat**	**Protein**
Corn starch	58.11	0	0
Powdered sugar	29.06	0	0
Vegetable shortening	0	75.12	0
Casein	0	0	87.17
DL-Methionine	0.11	0.2	0.11
AIN-76A vitamin mix	0.77	1.49	0.77
AIN-76A mineral mix	3.07	5.95	3.07
Choline chloride	0.18	0.34	0.18
Cellulose (alphacel)	8.72	16.91	8.72
Total caloric content	3.53 kcal/g	6.85 kcal/g	3.53 kcal/g

*Composition of diets are shown as % by weight.*

**TABLE 2:** Diet intake during acclimation days.

	**Diet intake (g)**		
**Acclimation day:**	**Carbohydrate**	**Fat**	**Protein**
Day 1	10.07 ± 0.11	10.13 ± 0.17	8.00 ± 0.50
Day 2	11.45 ± 0.24	11.4 ± 0.40	8.23 ± 0.84
Day 3	12.45 ± 0.44	12.47 ± 0.37	9.05 ± 0.66
Day 4	13.33 ± 0.90	12.23 ± 0.58	8.47 ± 0.50
Day 5	12.67 ± 1.22	12.5 ± 0.68	8.1 ± 0.67

*Intake of each individual diet in grams.*

### Experimental Design

All tests were started at the onset of the dark phase. For testing, rats were given the experimental treatment, followed by presentation of the three test diets in their home cage with *ad libitum* access to water throughout the experiment. Test diet intakes were then measured at 1, 2, 4, and 24 h after presentation of the diets. Immediately following the 24 h time point measurement, rats were returned to *ad libitum* chow feeding, and chow intake was measured 24 h later.

Rats were initially tested for the effects of acute fasting, followed by a test for the effects of cocaine 2 weeks later. For fasting, rats were tested in a counterbalanced order with half of the rats fasted for 24 h and the others having normal *ad libitum* access to standard chow in the 24 h preceding the first test day. The treatments were then reversed on the second test day 7 days later. For fasting, all food was removed at the onset of the dark phase 24 h prior to the start of the test, and the 3 test diets were provided in the home cage at the onset of the test 24 h later.

For cocaine treatment, rats were tested in a counterbalanced order with half of the rats receiving cocaine injection and the others receiving saline injection on the first test day. The injections were then reversed on the second test day 7 days later. Rats were injected intraperitoneally (i.p.) with 20 mg/kg cocaine or saline in a volume of 1.0 ml/1.0 kg body weight at the onset of the dark cycle and then returned to their home cage with the three test diets. The researcher measuring food intake was blind to the cocaine/saline treatments until the conclusion of the experiment.

### Data Analysis

All data are presented as mean ± SEM. Experiments were conducted using a within-subject design so that each rat received all treatments. A total of twelve rats were used in these experiments. In some cases, data for a specific test diet from an individual rat had to be excluded due to spillage of the entire food dish during one of the measurement periods. This also precluded calculation of the cumulative intake of that diet at the subsequent time points for this individual rat. When calculating the % decrease in intake following cocaine treatment and the relative increase in intake following fasting, data from some individual time points had to be omitted from the analysis due to little (< 0.1 g) or none of the individual diets being consumed during those time periods. Thus, for all analyses, the number of rats included ranged from 5 to 12 rats, with most analyses including a minimum of 7−8 rats. All data were analyzed with Microsoft Excel and statistical analysis was performed using IBM SPSS Statistics 25 or SigmaStat (v11.0, Systat Software, Inc.). A general linear model with repeated measures analysis was used with treatment (fed vs. fasted or cocaine vs. saline), diet (carbohydrate, protein, fat) and time as independent variables, followed by Sidak *post hoc* tests corrected for multiple comparisons. Paired *t*-tests were used to analyze post-test 24 h chow intake. A significance level was set at *p* < 0.05 *a priori* for all analyses.

## Results

**FIGURE 1:**
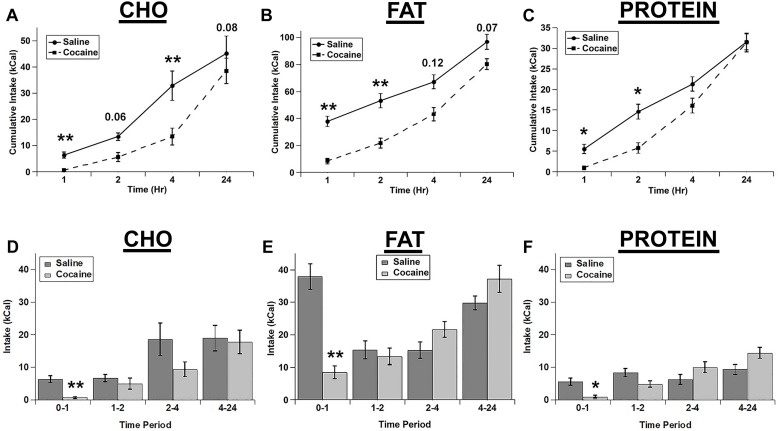
Cocaine inhibited all three test diets to a similar extent. Cumulative intake of the carbohydrate (CHO; **A)**, fat **(B)**, and protein **(C)** diets after saline and cocaine injection. Test diet intake during each individual time period (Non-cumulative) of the carbohydrate **(D)**, fat **(E)**, and protein **(F)** diets after saline and cocaine injection. Note the differences in the scale of the *Y*-axis for panels **A–C**. *n* = 7–12. ^*^p < 0.05; ^∗∗^*p* < 0.01.

We tested whether an i.p. injection of cocaine differentially affected the intake of the three individual macronutrient diets. Cocaine strongly decreased the intake of each diet with the total cumulative intake gradually returning nearly to control levels at 24 h post-injection (Figures 1A–C). For cumulative intake, there were significant main effects of drug (*F*(1,4) = 25.989; *p* = 0.007), diet (*F*(2,8) = 130.62; *p* < 0.001), and time (*F*(3,12) = 106.098; *p* < 0.001), and significant drug x diet (*F*(2,8) = 6.9, *p* = 0.018), diet x time (*F*(6,24) = 14.989; *p* < 0.001), and drug x diet x time (*F*(6,24) = 3.616, *p* = 0.011) interactions. The drug x time interaction was not significant (*F*(3,12) = 1.544; *p* = 0.254). *Post hoc* tests demonstrated that cocaine significantly inhibited the intake of all diets at 1 h and inhibited the cumulative intake of fat and protein at 2 h and carbohydrate at 4 h (Figures 1A–C).

We also analyzed the intake during each individual time period (Non-cumulative) to examine the timing of the effects of cocaine in more detail (Figures 1D–F). Analysis of the individual time periods revealed that cocaine only inhibited feeding of each diet during the 1st hour post-injection for each of the diets (Figures 1D–F). There were significant main effects of drug (*F*(1,4) = 50.043, *p* = 0.002), diet (*F*(2,8) = 41.623; *p* < 0.001), and time (*F*(3,12) = 12.9; *p* < 0.001), and significant drug x time (*F*(3,12) = 14.421; *p* < 0.001), diet x time (*F*(6,24) = 2.847; *p* = 0.031), and drug x diet x time (*F*(6,24) = 4.088; *p* = 0.006) interactions. In addition there was a trend for a drug x diet interaction, but this did not reach statistical significance (*F*(2,8) = 4.235; *p* = 0.056).

**FIGURE 2:**
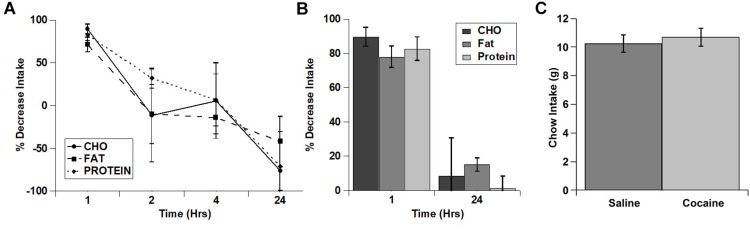
Cocaine inhibited the intake of all three test diets to a similar extent and did not alter post-test home cage chow intake. **(A)** % decrease in intake of each of the diets during each individual time period (Non-cumulative). **(B)** % decrease in cumulative intake of each of the diets at 1 and 24 h post-injection. **(C)** Post-test, 24 h home cage chow intake. *n* = 5–11 **(A,B)**. *n* = 12 **(C)**.

Rats ate much more of the fat diet under control conditions (see total kcal Intake in Figure 1), and visual examination of the data in Figure 1 raised the possibility that there may have been differences in the extent of inhibition of the intake of the diets. In order to examine this in more detail, we calculated the percent decrease in intake for each of the diets (Figures 2A,B). When the % decrease in intake was calculated for each individual time period there were no significant main effects and no significant diet x time interaction (diet: (*F*(2,2) = 3.021; *p* = 0.249, time: *F*(3,3) = 7.977; *p* = 0.061, diet x time interaction: *F*(6,6) = 2.605; *p* = 0.134). There was a trend toward a larger relative inhibition of the carbohydrate diet in the 1st hour when this was analyzed independently (Figure 2B), but this was not significant (*p* = 0.089). There were also no differences in the % decrease in cumulative intake at 24 h (Figure 2B). Finally, there were no differences in standard home cage chow intake in the 24 h period following the test (Figure 2C). Thus, it appears that cocaine rapidly inhibited the intake of each macronutrient to a similar extent and that this inhibition was relatively short lived (∼1 h).

**FIGURE 3:**
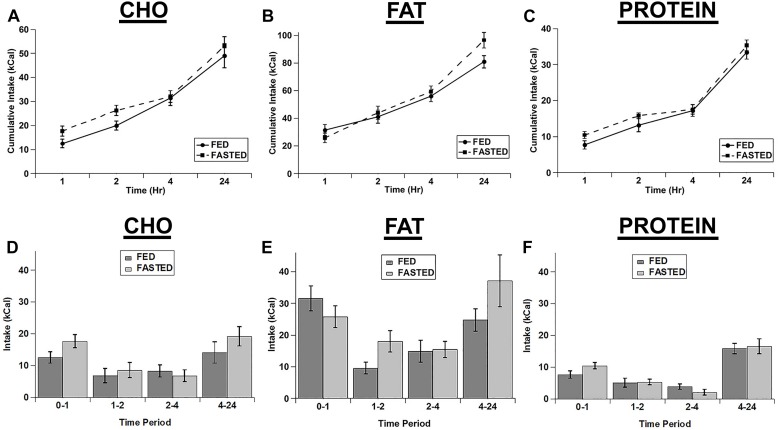
Fasting did not significantly increase the intake of any of the test diets. Cumulative intake of the carbohydrate **(A)**, fat **(B)**, and protein **(C)** diets in fasted and *ad libitum* fed rats. Test diet intake during each individual time period (Non-cumulative) of the carbohydrate **(D)**, fat **(E)**, and protein **(F)** diets in fasted and *ad libitum* fed rats. Note the differences in the scale of the *Y*-axis for panels **A–C**. *n* = 8–12.

We also tested whether an acute fast differentially increased the intake of the individual macronutrient diets. We initially calculated the cumulative intake of each of the test diets as was done for the cocaine injections (Figures 3A–C). Although there were significant main effects of diet (*F*(2,14) = 63.18; *p* < 0.001) and time (*F*(3,21) = 146.854; *p* < 0.001) and significant diet x time (*F*(6,42) = 13.003; *p* < 0.001) and feeding status x diet x time (*F*(6,42) = 2.321; *p* = 0.05) interactions, *post hoc* tests revealed no significant differences between fed vs. fasted rats at any times for any of the diets (Figures 3A–C). In addition the main effect for feeding status (*F*(1,7) = 4.323; *p* = 0.076) and the feeding status x diet (*F*(2,14) = 0.241; *p* = 0.789) and feeding status x time (*F*(3,21) = 2.462; *p* = 0.091) interactions were not significant. Similarly, when we analyzed each individual time point (Non-cumulative), there were main effects of feeding status (*F*(1,7) = 7.000; *p* = 0.033), diet (*F*(2,14) = 77.962; *p* < 0.001), and time (*F*(3,21) = 15.277; *p* < 0.001), but no significant interactions (feeding status x diet: *F*(2,14) = 2.243; *p* = 0.143; feeding status x time: *F*(3,21) = 1.777; *p* = 0.182; diet x time: *F*(6,42) = 1.829; *p* = 0.117; feeding status x diet x time: *F*(6,42) = 1.397; *p* = 0.238), despite apparent increases in the intake of the carbohydrate and protein diets at the 1 h time point (Figures 3D–F).

**FIGURE 4:**
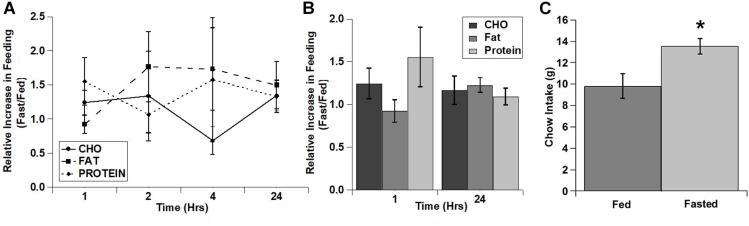
Fasting increased the intake of all three test diets to a similar extent and increased post-test home cage chow intake. **(A)** Relative increase in intake in response to fasting during each individual time period (Non-cumulative). **(B)** Relative increase in cumulative intake in response to fasting at 1 and 24 h post-injection. **(C)** Post-test, 24 h home cage chow intake. *n* = 8–12 **(A,B)**. *n* = 12 **(C)**. ^*^p = 0.007.

As with the cocaine injections, we also tested the relative increase in intake for each of the diets over time (Figures 4A,B). Despite apparent potential differences in the relative increase of the carbohydrate and protein diets vs. the fat diet in the 1st hour, there were no significant main effects and no significant interaction (diet: *F*(2,4) = 3.22, *p* = 0.147; time: *F*(3,6) = 0.934, *p* = 0.480; diet x time: *F*(6,12) = 0.896, *p* = 0.528). Interestingly, unlike the lack of effect on the test diets, fasted rats displayed a significant increase in chow intake during the 24 h after the conclusion of the diet choice test (Figure 4C).

## Discussion

In these studies, we tested whether acute fasting or cocaine injection differentially alters the intake of individual macronutrients using a three diet choice paradigm. Cocaine, which is a psychostimulant that has been widely shown to acutely decrease feeding, decreased the intake of all three diets (carbohydrate, fat, protein) to a similar level. There were robust decreases in feeding in the 1st hour following cocaine treatment, and a nearly complete return to control intake levels at 24 h post-treatment (Figure 1). There was a trend for cocaine to inhibit carbohydrate intake to a larger degree in the 1st hour post-treatment (relative the saline control condition) compared to fat and protein intake, but this did not reach statistical significance (Figure 2). Thus, cocaine appears to decrease the intake of each individual macronutrient to similar levels overall.

The ability of cocaine to inhibit all three test diets to a similar extent contrasts with the effects of a number of neuropeptides that appear to preferentially alter either fat or both fat and carbohydrate intake. For example, both galanin and neuropeptide Y (NPY) have been shown to preferentially increase the intake of carbohydrate and fat, depending on the rat’s initial diet preference (Smith et al., 1996, 1997). When injected into the paraventricular nucleus of the hypothalamus (PVN) or the amygdala, galanin selectively increased carbohydrate in carbohydrate preferring rats (Smith et al., 1997), but selectively increased both fat and carbohydrate intake in fat preferring rats (Smith et al., 1996). Similarly, injection of NPY into the PVN also selectively increased carbohydrate intake in carbohydrate-preferring rats and increased both carbohydrate and fat in fat-preferring rats (Smith et al., 1997). In this same study an acute fast also selectively increased fat intake in both carbohydrate- and fat-preferring rats Smith et al. (1997). Additional studies have also reported fat intake is preferentially decreased by both melanocortin-4 receptor agonists (Samama et al., 2003; Srisai et al., 2011), and dexfenfluramine, which decreases food intake through actions on serotonergic systems (Smith et al., 1998). Thus, these prior studies, some of which used a three diet choice test similar to that used here, have shown that multiple different manipulations altering food intake differentially affect the intake of the individual macronutrient diets. This is in contrast to the effects of cocaine observed here, however, with cocaine decreasing the intake of each diet to a similar extent. Thus, cocaine appears to globally decrease food intake in a non-selective manner.

Although the exact mechanism by which cocaine reduces food intake has not been identified, it is likely that it reduces feeding through its actions on dopamine. Amphetamine, which is another psychostimulant that reduces feeding, has been shown to decrease feeding primarily through its actions on dopamine circuits (Cannon et al., 2004). Thus, it seems likely that cocaine also inhibits feeding through actions on dopamine circuits. In light of this possibility, it is somewhat surprising that cocaine inhibited the intake of all three macronutrient diets to similar amounts. The rats clearly consumed the majority of their calories from the fat diet (see total caloric intake of each diet in Figure 1) and diets containing fat and sugar are highly rewarding and reinforcing. Therefore, it might be expected that the fat and/or carbohydrate (which is high in sugar) diets would be affected to a larger degree than the protein diet due to cocaine’s elevation of extracellular dopamine. This was not the case, however. Thus, it appears that either each of the individual test diets used here have equal rewarding and reinforcing qualities, or the anorexigenic effects of cocaine mediated by elevated dopamine are not due to changes in the rewarding or reinforcing qualities of the diets.

Cocaine also impacts multiple other neurotransmitters and neural circuits that play important roles in feeding. For example, cocaine also increases levels of norepinephrine and serotonin across much of the brain, including brain regions that are highly involved in the control of feeding, such as the hypothalamus. Furthermore, cocaine and amphetamine regulated transcript (CART) expression is also directly elevated following a single injection of cocaine or amphetamine, and CART has been shown to alter feeding (Douglass et al., 1995; Rogge et al., 2008). These hypothalamic circuits and many of the peptides produced within them also can interact with dopamine circuits to regulate feeding (Liu and Borgland, 2015; Roseberry et al., 2015; Rossi and Stuber, 2018). Thus, it is also possible that cocaine inhibits feeding through its actions on hypothalamic circuits and hypothalamic neuropeptides such as CART, either independently or through their concerted action with dopamine circuits. But, as described above, some of these neuropeptides and circuits differentially affect the intake of individual macronutrients (Smith et al., 1996, 1997, 1998), suggesting that these neuropeptides are not the main mediators of cocaine’s actions on feeding. Thus, more experiments are clearly required to identify the mechanisms by which cocaine alters food intake, including the equal inhibition of carbohydrate, fat and protein shown here.

In contrast to the effects of cocaine injection, an acute fast did not appreciably alter feeding of any of the three diets (Figure 3). There were slight increases in the intake of carbohydrate and protein in the 1st hour of refeeding (Figure 3), but there were no significant differences observed. Similarly, when comparing the relative increases in feeding (vs. the *ad libitum* fed control condition) there was a trend for increased intake of both carbohydrate and protein compared to fat in the 1st hour of refeeding, but this did not reach statistical significance. One potential explanation for the lack of effect of the acute fast is that the rats may have already been overconsuming the 3 choice diets under *ad libitum* conditions, so the acute fast was largely ineffective at driving intake even higher (i.e., a “ceiling effect”). The total 24 h caloric intake in *ad libitum* fed rats on the choice test was ∼160–175 kcal (See Figures 1–3), whereas the total 24 h caloric intake on the chow diet was ∼35–50 kcal (10–15 grams of 3.36 kcal/g chow). Thus, the rats could have already been overconsuming the test diets, resulting in the lack of an effect of the acute fast. Alternatively, the intermittent 3 diet choice paradigm could have altered the normal post-fast response independent of the amount of the diets eaten. Interestingly, there was an increase in normal chow intake in the 24 h time period immediately following 3 diet choice test in the fasted rats (Figure 4C), suggesting that the 3 diet choice feeding paradigm may have altered the normal post-fast feeding response, resulting in a delayed increase in intake when the rats were returned to a normal chow diet. The lack of effect of the acute fast also is in contrast to a prior study that used a similar approach to examine whether fasting differentially alters individual macronutrient intake (Smith et al., 1997). In the study by Smith et al. (1997), the rats were constantly exposed to the 3 diets, both on test days and the intervening test days, with no access to normal chow during the entire test. In our studies, the rats were only exposed to the 3 choice diets on acclimation and test days, however. Thus, although we acclimated the rats to the 3 diet choice paradigm extensively until they had a stable intake of each of the diets (Table 2), it appears that this paradigm may have altered the rats post-fast response to the diets, either due to a ceiling effect or through some other unknown mechanism, and resulted in a delay in the post-fast hyperphagia until they had access to normal chow on the following day. It should also be noted that the fasting and *ad libitum* fed control test days were counterbalanced so only half of the rats were fasted on a single test day, which precluded any adverse events on an individual test day from influencing these responses. Thus, although there were not robust responses to the acute fast, it does not appear that fasting differentially alters the intake of individual macronutrients.

Although these studies have provided novel and important information on how different perturbations of feeding affect the intake of individual macronutrients, there are some limitations to these studies. First, the use of a three diet choice test, with each diet containing a single macronutrient (with added vitamins and minerals), could have altered the normal response of the rats to cocaine and/or fasting. As described above, this appears to be true for the effects of fasting, as the rats did not show significant elevations in the intake of any of the test diets, but did increase their chow intake 24 h later. This suggests that the use of the individual test diets may have altered the rats’ normal post-fast feeding response, either due to their elevated caloric intake during exposure to the test diets or through some other unknown mechanism. Cocaine caused robust decreases of each test diet, however, showing that the three diet choice test does reflect the normal alterations in feeding for some feeding-altering stimuli. Furthermore, the use of diets containing individual macronutrients does not reflect the normal feeding of animals or humans in the wild, where nearly all foods are complex combinations of different amounts of macronutrients and micronutrients. Another limitation of this study was that only male rats were included in these experiments. Males and females have been shown to differ in their feeding and weight responses to various genetic and pharmacological manipulations (Asarian and Geary, 2013). Thus, it is possible that female rats may show a different response to cocaine or fasting compared to the male rats used here, but future studies will be required to test this possibility.

Another potential issue with these studies is that only a single, non-contingent injection of a high concentration of cocaine (20 mg/kg) was used in these experiments. It is possible that rats could show differential sensitivity to the effects of lower doses of cocaine for the individual diets that were lost when feeding was inhibited with a maximum dose. Cocaine has sensitizing effects on locomotion and other behaviors when given repeatedly, however, which could complicate the interpretation of studies using multiple injections of cocaine in the same animal. Future studies should test additional, lower doses of cocaine in separate rats, however, to ensure that it does not have differential effects on the individual diets at lower doses. It is also possible that the stress of the acute, non-contingent injections or the enhanced locomotor activity following cocaine could have influenced these results. Rats were tested in a counterbalanced order, with half of the rats receiving cocaine on the first test day and the other half receiving cocaine on the second test day, so it appears unlikely that the stress of the initial injection influenced the effects of cocaine in these studies. Furthermore, when the relative changes in intake with cocaine and fasting were compared between rats receiving cocaine/fasting on the first vs. second test day there were no significant differences for any of the diets (*p* > 0.29 for all tests). Thus, it does not appear that stress or other side-effects associated with the single cocaine injection confounded the results of these studies. It is possible that the increased locomotor activity following cocaine could have influenced the reduction in intake, but it does not appear that this would influence the interpretation of these results, as this would be expected to affect each diet equally.

In summary, we have shown that cocaine strongly inhibited the intake of each macronutrient to a similar degree, whereas an acute fast showed only marginal increases in the intake of each diet, with no differences between the intakes of each individual macronutrient. Thus, these studies have significantly advanced our understanding of the central mechanisms controlling feeding.

## Data Availability

The raw data supporting the conclusions of this manuscript will be made available by the author, without undue reservation, to any qualified researcher upon request.

## Ethics Statement

The animal study was reviewed and approved by Georgia State University Institutional Animal Care and Use Committee.

## Author Contributions

AR designed the experiments. NM and AR performed the experiments and analyzed the data. NM collected all the data and wrote an initial draft of the “Materials and Methods” and “Results” sections, and AR edited these sections and wrote the rest of the manuscript.

## Conflict of Interest Statement

The authors declare that the research was conducted in the absence of any commercial or financial relationships that could be construed as a potential conflict of interest.
